# Spaghetti Meat Abnormality in Broilers: Current Understanding and Future Research Directions

**DOI:** 10.3389/fphys.2021.684497

**Published:** 2021-05-31

**Authors:** Giulia Baldi, Francesca Soglia, Massimiliano Petracci

**Affiliations:** Department of Agricultural and Food Sciences, University of Bologna, Cesena, Italy

**Keywords:** broilers, spaghetti meat, spaghetti breast, growth-related abnormalities, histology, meat quality, causative mechanisms

## Abstract

Spaghetti meat (SM) is a recent muscular abnormality that affects the *Pectoralis major* muscle of fast-growing broilers. As the appellative suggests, this condition phenotypically manifests as a loss of integrity of the breast muscle, which appears soft, mushy, and sparsely tight, resembling spaghetti pasta. The incidence of SM can reach up to 20% and its occurrence exerts detrimental effects on meat composition, nutritional value, and technological properties, accounting for an overall decreased meat value and important economic losses related to the necessity to downgrade affected meats. However, due to its recentness, the causative mechanisms are still partially unknown and less investigated compared to other muscular abnormalities (i.e., White Striping and Wooden Breast), for which cellular stress and hypoxia caused by muscle hypertrophy are believed to be the main triggering factors. Within this scenario, the present review aims at providing a clear and concise summary of the available knowledge concerning SM abnormality and concurrently presenting the existing research gaps, as well as the potential future developments in the field.

## Introduction

About 10 years ago, the appearance of a novel cluster of muscular abnormalities affecting the *Pectoralis major* muscle of fast-growing broilers has alarmed the poultry industry. From there on, a growing body of literature has been focused on the etiology behind their appearance, implications on meat quality, as well as the attempts to mitigate their occurrence and narrow down the negative perception related to animal welfare (Griffin et al., 2018; Petracci et al., 2019; Baldi et al., 2020a). Within the industry vernacular, these myopathies are commonly called White Striping (WS), Wooden Breast (WB), and Spaghetti Meat (SM), each of which owning peculiar and distinctive traits from which their names originated. The first is indeed characterized by the occurrence of white lines running parallel to the muscle fibers on the surface of *P. major* muscle (Kuttappan et al., 2013b); the second manifests as a severe hardening of the pectoral muscle (Sihvo et al., 2014), while the third displays as a loss of integrity of the muscle fiber bundles composing the breast muscle itself, which appears mushy and sparsely tight (Baldi et al., 2018). Due to their alarming incidence rates and outstanding impact on meat quality, a large number of studies has been published on WS and WB abnormalities over the years, focusing on the understanding of their etiology, the molecular pathways potentially responsible for their occurrence as well as their implications on eating, nutritional, and technological properties of meat (Petracci et al., 2019). On the contrary, the information regarding SM is today still limited and only few studies have been published since its appearance (Table 1). Thus, the present review aims at providing a clear and succinct summary of the available knowledge concerning SM condition and presenting the current research challenges as well as the potential future developments in the field.

**TABLE 1:** Chronological sequence of available published studies concerning spaghetti meat condition and relative addressed topics.

			Examined traits					
			
References	Implications of genetic and environmental factors on incidence levels	Detection tools	Meat processing solutions	Histology	Proximate composition	Protein and/or amino acid profile	Collagen	Technological quality
Sirri et al., 2016	🌑							
Baldi et al., 2018			🌑	🌑	🌑	🌑		🌑
Córdova-Noboa et al., 2018	🌑							
Zampiga et al., 2018	🌑							
Baldi et al., 2019			🌑		🌑	🌑	🌑	🌑
Montagna et al., 2019	🌑							
Soglia et al., 2019a			🌑			🌑		🌑
Mazzoni et al., 2020				🌑			🌑	
Bailey et al., 2020	🌑							
Campo et al., 2020		🌑						🌑
Morey et al., 2020		🌑						
Pascual et al., 2020	🌑							
Soglia et al., 2020						🌑	🌑	
Tasoniero et al., 2020					🌑	🌑	🌑	🌑
Yu et al., 2020								🌑
Pascual et al., 2021					🌑			🌑
Sanden et al., 2021				🌑			🌑	
								

## Up-To-Date Knowledge

### Morphological Characteristics and Incidence Levels

**FIGURE 1:**
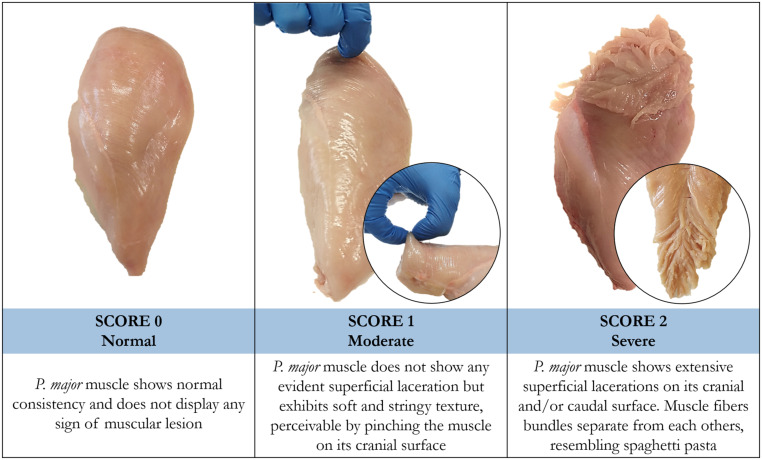
Representative images of chicken breast fillets with normal (score 0), moderate (score 1) and severe (score 2) degrees of Spaghetti Meat.

Spaghetti Meat defect was first recounted in 2015 with the name of “Mushy Breast” and described as a myopathy causing the loss of muscle integrity of the *P. major* muscle of fast-growing chickens (Bilgili, 2015). Later on, this condition has been commonly recognized with the name of “Spaghetti Meat” or “Spaghetti Breast” since, as the appellative suggests, it phenotypically manifests with the detachment of the fiber bundles composing the pectoral muscle, which appears soft, mushy, and sparsely thigh, resembling spaghetti pasta. Since the severity of SM defect might be variable, Sirri et al. (2016) proposed a classification criterion based on a three-score scale. Indeed, depending on the severity score, the occurrence of SM can be detected either palpably, due to the soft and stringy structure perceived by pinching the muscle on its surface, or visually, due to the expanded superficial lacerations (Figure 1). As will be further explained (see section “Implications on meat quality and practical solutions”), meats severely affected by SM are usually downgraded and incorporated into the formulation of further processed products, while moderate cases can be marketed for fresh retailing. Generally, SM condition mostly manifests in the ventro-cranial portion of the fillet, but there is growing evidence that also the caudal section and, occasionally, leg muscles might be affected as well. Recently, a sporadic occurrence of SM defect has been also signaled on the pectoral muscles of commercial turkeys, even though no information is yet available concerning possible similarities with the same condition reported for broilers (Zampiga et al., 2020).

Data concerning the incidence rates of SM condition are limited and sometimes contradictory, likely due to both the variation of classification criteria among the abattoirs and the complication related to the concurrent presence of other myopathies within the same muscle, since SM can be comorbid with WB and, more likely, with WS (Baldi et al., 2018; Bailey et al., 2020). However, an Italian survey carried out on 16,000 breasts reported that around 21% of samples were affected by SM (Baldi et al., 2020a), while a Brazilian study evaluated about 10% of SM fillets on a total of 5,580 breasts (Montagna et al., 2019). While it has been widely recognized that the incidence rates of WS and WB significantly upsurge with higher growth rate, breast yield as well as body weight and age at slaughter of birds (Kuttappan et al., 2013a, 2016; Papah et al., 2018), this trend has not been fully confirmed for SM. However, Pascual et al. (2020) intriguingly found significantly higher rates of SM in females rather than males (25.0% vs. 3.1%, respectively), contrarily to what observed for WS and WB, whose incidence levels were found to be greater in males regardless the slaughter weight (Lorenzi et al., 2014; Trocino et al., 2015). To the best of our knowledge, the reasons for the higher incidence of SM condition in female individuals are still unknown, albeit a recent study highlighted an increased expression of genes related to connective tissue proliferation in male broilers, making them more prone than females to develop WB condition (Brothers et al., 2019). However, a possible role of a different hormonal response (e.g., insulin growth factor, somatotropic hormone, myostatin, etc.) regulating metabolism, protein synthesis, patterns of intramuscular fat deposition, and intracellular signaling during the development of myopathic conditions might be also speculated.

### Histological Traits

Microscopic investigations performed on SM muscles highlighted several histologic features commonly reported also for WS and WB, such as nuclei internalization, inflammatory cells infiltration, necrosis, fiber lysis, and concurrent presence of small regenerative fibers combined with abnormal ones showing larger diameter (Baldi et al., 2018). On the other hand, the distinctive microscopic feature of SM condition is the progressive rarefaction of the endomysial and perimysial connective tissue, that likely leads to the detachment of the muscle fibers from each others (Baldi et al., 2018). Moreover, histological observations highlighted the presence of small and thin fibers, interspersed by immature/newly deposited connective tissue (Baldi et al., 2018; Sanden et al., 2021). The presence of these fibers, distinguished by a remarkably reduced cross-sectional area, suggests the ongoing of regenerative processes taking place in the muscle, as a natural response mechanism to cell necrosis (Mazzoni et al., 2020). However, a different phase of cell regeneration can be hypothesized when comparing SM and WS samples’ immunoreactivity to vimentin, suggesting a different progression of the regenerative processes (Soglia et al., 2020). A recent study also investigated the distribution of collagen type III and its precursor (i.e., procollagen type III) in SM samples since, being involved in fibrillogenesis, their possible altered deposition might have a key role in the occurrence of muscular abnormalities. In this regard, the findings highlighted an altered immunoreactivity to procollagen type III in SM samples, suggesting a compromised collagen turnover and synthesis (Mazzoni et al., 2020).

### Implications on Meat Composition

The degenerative processes and repair mechanisms taking place in the muscles affected by growth-related abnormalities (e.g., inflammatory processes, necrosis, fibrosis, lipidosis, etc.) inevitably alter the proximate composition of meat and, as a direct consequence, negatively affect its nutritional value (Petracci et al., 2019). In detail, as reported by Baldi et al. (2018, 2019), the occurrence of SM is associated with a remarkable relatively reduction of protein content (-10%, when compared to unaffected muscles) coupled with a concurrent increase in fat and moisture levels (+21.8 and + 3.0%, respectively). The same authors reported a lower content of EPA and DHA in meat affected by SM, likely accountable for a different expression of the genes encoding for Δ5 and Δ6 desaturases, as previously hypothesized by Soglia et al. (2016a). As concern mineral profile, a study carried out by Tasoniero et al. (2020) reported greater calcium and sodium levels in SM samples if compared to unaffected ones, speculating a possible connection between cation homeostasis disturbances and the appearance of pathological mechanisms leading to cell injury development. Intriguingly, the analysis of the available literature highlighted that SM samples possess analogous amounts of total and soluble collagen of unaffected breasts (Baldi et al., 2018; Campo et al., 2020; Tasoniero et al., 2020). On the other hand, Baldi et al. (2019) found lower hydroxylysylpyridinoline contents in muscles affected by SM, suggesting a feeble collagen cross-linking (i.e., presence of immature collagen) and corroborating what found from previous histological observations (see section “Histological traits”). Accordingly, following investigations through polarized FTIR-spectroscopy, Sanden et al. (2021) reported that SM muscles possess thin, loose and immature collagen fiber bundles, which are also poorly packed. The same authors also mentioned that SM shows a higher amount of glycosaminoglycans in perimysial connective tissue, corroborating the hypothesis put forward by Papah et al. (2018) of a shift in glucose flux toward the synthesis of molecules composing the extracellular matrix in myopathic muscles.

### Implications on Meat Quality and Practical Solutions

Albeit only few studies have been published concerning the implications of SM condition on meat quality (see Table 1), its occurrence seems to affect meat technological properties and functionality to a lesser extent if compared to WS and WB (Baldi et al., 2019; Pascual et al., 2021). Overall, the occurrence of SM is associated with higher meat yellowness and ultimate pH values (Baldi et al., 2018; Soglia et al., 2019a; Tasoniero et al., 2020). While Yu et al. (2020) did not find any difference in water holding capacity assessed on fresh, frozen, and cooked SM samples, Pascual et al. (2021) reported greater drip and cooking losses in SM samples compared to normal ones. The latter result corroborates the outcomes obtained through time-domain nuclear magnetic resonance analysis, which evidenced increased amounts of bound and extra-myofibrillar water fractions in SM fillets (Soglia et al., 2019a). As suggested by the longer relaxation times, the abovementioned water fractions are concurrently less tightly bound to the muscle tissue, highlighting an altered water distribution and mobility within SM muscles and, thus, an impaired ability to hold constitutional water (Baldi et al., 2019). This hypothesis is further supported by the results obtained by Tasoniero et al. (2020), who found a remarkable reduction of myofibrillar protein solubility, salted-induced water uptake, final yield as well as emulsion stability in SM fillets if compared to unaffected ones. Intriguingly, SM breasts did not display any upsurge in protein carbonylation levels (Baldi et al., 2018; Soglia et al., 2019a), suggesting that the reduced water holding capacity is not imputable to the ongoing of oxidative processes occurring at the expenses of muscular proteins. Thus, overall, this phenomenon can be explained by further mechanisms which essentially share the same underpinning factor. Precisely, the complete re-organization of muscle architecture following myodegeneration and necrosis leads to a reduction in the number of functional myofibrils and, therefore, to a lower potential of the muscle to bind water (Soglia et al., 2016a; Tasoniero et al., 2017). Indeed, the impaired water holding capacity of SM samples might be the direct consequence of their reduced protein solubility, probably due to the ongoing of protein degradation processes as evidenced by both the higher myofibrillar fragmentation index of SM muscles if compared to unaffected ones (Baldi et al., 2018; Tasoniero et al., 2020) and the greater concentration of free amino acids deriving from the breakdown of muscular proteins (Soglia et al., 2019a). In support of this, electrophoretic analysis evidenced an increased number of high molecular-weight bands in SM samples, presumably originating from the degenerative processes associated with the occurrence of muscular abnormalities (Baldi et al., 2018).

As concern texture, compression forces obtained for raw meat did not evidence any difference between SM samples and their unaffected counterparts (Baldi et al., 2019). However, after the cooking process, SM fillets showed a softer texture following compression, Meullenet-Owens Razor Shear as well as Allo–Kramer tests (Baldi et al., 2019; Pascual et al., 2021), probably as a result of their reduced collagen cross-linking degree. In addition, a number of studies have been carried out to investigate whether SM lesions display intra-fillet variations, revealing that the occurrence of this condition mainly alters meat composition and quality traits of the superficial layer of the muscle, while the deep one is almost unaffected (Baldi et al., 2019; Tasoniero et al., 2020; Yu et al., 2020). However, to the best of our knowledge, any published study specifically speculates about those divergences. Nevertheless, intensive selection practices carried out over years permitted to accomplish a remarkable development of the breast muscle, so much so that the impressive thickness of its cranial section might compress the pectoral artery, reducing muscular oxygenation (Soglia et al., 2021). Thus, a feasible hypothesis for the abovementioned intra-fillet variations may rely on a different blood flow between the surface of the muscle, phenotypically exhibiting the defect, and its deep counterpart. The first, being thicker and likely less oxygenated compared to the latter, might be more prone to develop inflammatory processes due to the weakened blood supply and impaired displacement of metabolic end-products, thus resulting in the advancing of hypoxic conditions which are believed to be the triggering factor for the manifestation of growth-related abnormalities (Abasht et al., 2016; Malila et al., 2019; Greene et al., 2020). Indeed, as observed at least for WS and WB, the severity of the histological lesions gradually weakens when moving from the antero-ventral to the antero-dorsal section of *P. major* muscle (Soglia et al., 2016b; Clark and Velleman, 2017; Baldi et al., 2020b).

The abovementioned detrimental effects of SM condition on meat quality may account for economic losses related to decreased yields during processing. Generally speaking, meats severely affected by SM are usually downgraded and incorporated into the formulation of further processed products, while moderate cases can be marketed for fresh retailing (Petracci et al., 2019). However, the growing consumers’ demand for thin-sliced chicken breast meat has made the occurrence of SM particularly challenging for the processing industry since, being SM often detectable only after the slicing process, the amount of downgraded/discarded meat could be even exacerbated. Thus, being the in-line detection of abnormal meat a relevant matter especially for the meat packing plants, Morey et al. (2020) recently proposed the feasibility of bioelectrical impedance analysis to identify SM breasts, while Campo et al. (2020) suggested the analysis of color reflectance as a possible tool to detect those breasts affected by any degree of WS and SM in the processing line. Aside from this, it is noteworthy to mention that SM myopathy cannot be noticed in the living animal, contrarily to WB condition whose presence can be detected by palpating the breast of the live bird and lifting up its wings to assess the ability to achieve back-to-back wing contact (Hosotani et al., 2020). This might represent a useful tool for breeder companies to exclude birds showing WB defect from the pedigree lines, even though this would entail the possible risk to indirectly select individuals showing SM condition. Indeed, evidence suggests that a reduction of WB occurrence in some flocks is sometimes associated with a concurrent increase in SM condition levels.

Thus, given the detrimental impact of SM condition and, more generally, of growth-related abnormalities on chicken meat quality and the economy of poultry meat producers, the pursuit for potential solutions to mitigate these undesirable implications is calling the attention of the scientific community and have been recently summarized in the reviews written by Barbut (2019), Petracci et al. (2019), and Baldi et al. (2020a). Considering the current lack of efficient animals’ nutrition and management strategies to reduce SM defect without affecting slaughter performances (Sirri et al., 2016; Córdova-Noboa et al., 2018; Zampiga et al., 2018), to date, the most promising approach seems to develop processing solutions aimed at reducing the implications on the final quality of meat. Within this context, the most practical solution so far is represented by the separation of the superficial and deep sections of the fillets, addressing the first for the manufacture of processed products, while the latter for fresh retailing. Indeed, as reported in the previous paragraph, the occurrence of SM negatively impacts only the surface of the muscle, thus allowing to downgrade a reduced portion of the fillet. As previously suggested for WB (Brambila et al., 2017; Xing et al., 2017; Chen et al., 2018), downgraded meat could be then included in the formulation of ground or finely comminuted meat products, where the addition of functional ingredients (i.e., starches, phosphates, hydrocolloids, etc.) could mask its impaired technological properties (Petracci et al., 2019). Moreover, since it has been reported that freezing – and subsequent thawing – do not result in any further worsening of SM meat quality traits (Soglia et al., 2019a), downgraded fillets can be frozen and afterward included in the formulation of processed products, guaranteeing a greater flexibility for the poultry processing industry.

## Current Challenges and Potential Future Developments

Considering the recentness of SM condition, the mechanisms underpinning its appearance are still partially unknown and less investigated compared to WB and WS. However, the same histological alterations shared among WB, WS, and SM fillets suggest a common network of causative mechanisms responsible for their occurrence, in which cellular stress and hypoxia – caused by muscle hypertrophy induced by selection – play a key role (Sihvo et al., 2018; Young and Rasmussen, 2020; Soglia et al., 2021). Considering their common histological traits, one of the most compelling challenges for the scientific community is to determine the reason why growth-related myopathies phenotypically manifest differently. Within this context, Soglia et al. (2020) recently speculated that the distinctive phenotype of WB, WS, and SM meat could be associated, at least partially, to the synthesis of vimentin, a protein considered a marker of muscle fiber regeneration and directly involved in the coordination of fibroblast proliferation (Cheng et al., 2016). In more detail, it has been suggested that the lack of correspondence between the up-regulated gene encoding for vimentin and its encoded protein found in SM muscles might be responsible for an altered distribution of fibroblasts in the perimysial compartment, which would ultimately result in a progressive rarefaction of the connective tissue, a typical trait of SM myopathy (Soglia et al., 2020).

Otherwise, the distinctive phenotype of SM has been also thought to be associated with an excessive build-up of lactic acid in the muscle, leading to the inhibition of protein synthesis regulating collagen development (Anton et al., 2019). On the other hand, contrarily to what previously observed for WS and WB (Radaelli et al., 2017), it must be pointed out that the low heritability levels of SM may indicate the impact of non-genetic factors on the variance of the myopathy traits (Bailey et al., 2020). Indeed, the weak correlation between the incidence of SM condition and animals’ growth rate, age, and weight at slaughter might hint at other “contributing factors” related to slaughtering operations (e.g., scalding, de-feathering, chilling, post-mortem deboning time and technology, etc.) which can exploit a role in the manifestation of SM condition or, at least, in the worsening of its severity level. Indeed, a recent technical report highlighted that the incidence of SM was found to be up to 50% higher in carcasses subjected to slow carcass chilling compared to their fast-cooled counterparts (Anton et al., 2019). The authors justified this phenomenon with an excessive accumulation of lactic acid in the muscle when the carcass is still warm. This would stimulate the activity of proteases responsible for the degradation of connective tissue and subsequent excessive softening of the meat (Anton et al., 2019). However, from the analysis of the available literature, no research aimed at establishing the effect of chilling conditions on the occurrence of SM is available yet, thus being a stimulating starting point for future investigations. Another appealing challenge for the poultry processors could be the understanding of the possible role of scalding and plucking procedures in a further worsening of the consistency of those fillets exhibiting a mild level of muscle destructuration. Indeed, given the presence of weakened perimysial connective tissue in SM samples (Baldi et al., 2018), it could be hypothesized that scalding temperatures combined with an aggressive de-feathering could likely worsen an already compromised muscular structure, making the muscle fibers more prone to be torn apart during handling and fileting. In addition, a new emerging quality issue phenotypically resembling SM condition was recently found to affect the *P. minor* muscles of broiler chickens. This defect is named “gaping” and has been examined for the first time in the study of Soglia et al. (2019b), who suggested that its occurrence is especially related to peri-mortem factors as well as slaughtering procedures. Notwithstanding, further studies should be performed to establish whether a relationship exists between the occurrence of gaping defect in the *P. minor* muscles and SM in their corresponding *major* counterparts.

In conclusion, considering the available knowledge and the existing research gaps about SM myopathy, the focus of future investigations might be directed into the unraveling of the role of peri-mortem procedures in the development of this condition, as well as the underpinning factors that make the incidence of SM higher in female individuals and inversely correlated to the manifestation of WB condition.

## Author Contributions

GB was the primary writer and performed most of the literature search. FS and MP supported the redaction of all sections. All authors listed have made a substantial, direct and intellectual contribution to the work, and approved it for publication.

## Conflict of Interest

The authors declare that the research was conducted in the absence of any commercial or financial relationships that could be construed as a potential conflict of interest. The reviewer EP declared a past co-authorship, with one of the authors, FS, to the handling editor.
